# Medical school origins of award-winning psychiatrists; analysis of a complete national dataset

**DOI:** 10.1186/s12909-024-05135-5

**Published:** 2024-03-01

**Authors:** Sinclair Steele, Gabriel Andrade, Jigna Stott

**Affiliations:** College of Medicine, Ajman University, University Street, Al Jerf 1, Ajman, United Arab Emirates

**Keywords:** Psychiatrists, Medical schools, Medical careers, Award-winners, Globalization, International Medical Graduates

## Abstract

**Background:**

Britain attracts doctors from all over the world to work in the National Health Service. Elucidating the educational backgrounds of award-winning doctors working in the country is potentially an important medical education issue and a merit award audit. Using the British clinical merit award schemes as outcome measures, we identify medical school origins of award-winning doctors who have been identified as having achieved national or international prominence.

**Methods:**

The Clinical Excellence Awards/Distinction Awards schemes select doctors in Britain who are classified as high achievers, with categories for national prominence and above. We used this outcome measure in a quantitative observational analysis of the 2019–20 dataset of all 901 award-winning doctors. Pearson's Chi-Square test was used where appropriate.

**Results:**

Five university medical schools (London university medical schools, Glasgow, Edinburgh, Aberdeen and Cambridge) accounted for 59.1% of the psychiatrist award-winning doctors in the 2019 round, despite the dataset representing 85 medical schools. 84.1% of the psychiatrist award-winners were from European medical schools, compared to 92.1% of the non-psychiatrist award-winners. International medical graduates accounted for 22.7% of the award-winning psychiatrists. Psychiatrists with the lower grade national awards came from a more diverse educational background of 17 medical schools. IMGs represented diverse medical schools from five continents and were most represented in the lowest grade of national merit awards at 24.2%.

**Conclusions:**

The majority of the award-winning psychiatrists originated from only five medical schools. A greater diversity of medical school origin existed for the lowest grade national psychiatrist award-winners. International medical graduates contributed substantially to these award-winners; psychiatrist award-winners were more likely to be international medical graduates (22.7%) than non-psychiatrist award-winners (10.8%). This study not only indicates educational centres associated with the production of award-winners but also provides students with a roadmap for rational decision making when selecting medical schools.

## Background

The backbone of any good clinical practice is access to high quality psychiatric specialists whose intervention is essential to manage patients in acute mental crisis or experiencing chronic psychiatric illness; a truism that has been brought into sharp relief by the preceding 30 months of the COVID-19 pandemic. Britain is unique in having longstanding national merit award systems that reward doctors who are deemed to be performing well. Using these awards as an outcome measure and identifying the educational characteristics of such clinicians facilitates our understanding of the best way to create more of these high-achieving doctors. Our project examines the educational backgrounds of these successful psychiatric clinicians.

Historically, in Britain there have been two clinical national merit award schemes in place to reward successful clinicians working in the National Health Service (NHS), the Clinical Excellence Awards Scheme (covering Wales and England) and the Distinction Awards Scheme (covering Scotland) [1]. Although the Scottish scheme is continuing, the Clinical Excellence Award scheme is currently being iteratively improved and renamed as the National Clinical Impact Awards (NCIA). The doctors in receipt of any of these awards benefit not only from the positive reputational and career effects but also from the recurring and explicitly financial incentives associated with such honours [1].

Although these award schemes were originally established after World War II for the purpose of encouraging senior doctors to support the newly-formed NHS, the awards have been recurring subjects of discussion amongst the medical community. Accordingly, the process by which merit awards have been assigned has long been a source of spirited debate. Consequently, they have been analyzed with regard to award objectivity [2], distribution by specialty [3] by region [3], by gender [1] by age [4] and by ethnicity [5] but *not by medical school of origin*. Such constructive criticism has led to iterative improvements in the award schemes over the last three decades. Many commentators agree that some system should be in place to reward successful clinicians [6] and these awards are viewed as national measures of clinical career success—accounting for their continued utility more than 60 years after their inception. This original study is intended to be part of our series of articles that adds to the educational discussion by relating the psychiatrist and non-psychiatrist award-winners to their *medical schools of origin*. We place our findings in the context of educational, demographic and career implications for medical students and doctors aspiring to achieve career success [7, 8].

## Methods

The lists of the psychiatrist award-winners and non-psychiatrist award-winners were retrieved from the source material of the Scottish Distinction Awards (DA) Report for 2019–2020 [9] together with the (England and Wales) Clinical Excellence Awards (CEA) Annual Report for 2019–2020 [10] covering the 2019–2020 awards round. These lists included both the new awardees and the previous award-winners who continued to hold their awards. The originating medical schools were found by using the published Medical Register, UK [11] as well as the published Dental Register, UK [12, 13]. The total number of award-winners was 901—the university medical schools of origin were successfully identified for 99.8% of these clinicians [13]. Accordingly, 899 doctors were included in the analyzed dataset. Award-winning doctors in the publications above, who were designated as specializing in any of the psychiatric disciplines, were included in this study [13]. In the 2019–20 award round the following specialties were specified in the databases: forensic psychiatry, general psychiatry, psychiatry (non-specific) and psychiatry of learning disability [13].

The rankings of medical schools by number of merit award-winning alumni were determined by summation of the number of psychiatrist award-winners of A plus (A^+^), A or B grade (or platinum, gold, silver or bronze award-winners) [13]. Only these national level Clinical Excellence Awards and Distinction Awards were included in this study [13]. Combining these parallel and similar award gradings permitted all of Britain's (England, Wales and Scotland) excellence award-winners to be analyzed together [13]. As part of our analysis of the grades of awards we combined the award categories to explicitly show the three tiers of national merit awards; A plus and platinum award-winners were combined to yield the top tier (tier 1) of national psychiatrist awards [13]. The A and gold awards were combined to create the intermediate tier (tier 2) of national psychiatrist awards [13]. Finally, the B and silver/bronze awards were combined to create the lowest tier (tier 3) of national psychiatrist merit awards [13]. The same approach was taken with the non-psychiatrist data [13].

The rankings of the medical schools by the number of merit award-winning alumni were approximately size corrected by dividing the total number of award-winners that were alumni of the medical school by the number of admissions to the undergraduate medical school in the 2019–20 academic year [13]. We used this pragmatic approach to estimate the size correction rather than the more ideal but inaccessible integral of medical school graduation numbers against time for approximately the last 50 years [13]. The comparison of the distributions of award-winners (psychiatrist merit award-winners versus non-psychiatrist merit award-winners) was quantified using Pearson's Chi-Square test with the significance level set to *p* < 0.01 [13]. The variables used here were (i) Medical school of origin and (ii) (Binary) psychiatrists vs non-psychiatrists. The variables used for the Chi-Square test on the continents of origins data were (i) Continent of origin (ii) (Binary) psychiatrists vs non-psychiatrists. All procedures were performed in compliance with the pertinent guidelines [13].

The methods that were applied in our study, and that cover the description in this methods section, were similar to and closely derived from an earlier publication in this series, which we cite here [13]. No ethical/access approvals were required for this retrospective analysis of published publicly available data of non-clinical origin.

## Results

The 899 doctors from the 2019–20 round of national merit award-winners, comprised 44 psychiatry clinicians. 91% were classified as either non-specific "psychiatrists" or "general psychiatrists." As a proportion of all the national merit award-winners in the 2019–20 round, the psychiatrists only accounted for 4.90%. By way of comparison, the surgeon merit award-winners comprised 25% of the total number of national merit award-winners in the 2019–20 round—more than five times as many as the psychiatrists.

Table 1 shows the ten medical schools that attained the greatest number of merit award-winners; these clinicians possessed platinum, gold, silver, bronze, A plus, A or B awards. Graduates of London university medical schools, Glasgow, Edinburgh, Aberdeen and Cambridge medical schools accounted for 59.1% of all the national merit awards held by psychiatrists. If all the psychiatrist award-winners are considered (and not only the medical schools from which the majority of the award-winners originate) then the medical school rankings by number of award-winners is: (1) London (2) Edinburgh (3) Glasgow (4) Aberdeen (5) Leeds (6 =) Cambridge and (6 =) Malta.

**Table 1:** Top 10 medical schools; analysis by number of psychiatrist award-winners, number of non-psychiatrist award-winners and total number of award-winners as of 2019–2020

Medical school	Total number of award-winners	Number of psychiatrist award-winners	Percentage of psychiatrist award-winners	Number of non-psychiatrist award-winners	Percentage of non-psychiatrist award-winners
London	179	9	20.45	170	19.88
Glasgow	113	5	11.36	108	12.63
Edinburgh	84	6	13.64	78	9.12
Aberdeen	60	4	9.09	56	6.55
Oxford	45	0	0.00	45	5.26
Cambridge	43	2	4.55	41	4.80
Manchester	38	0	0.00	38	4.44
Birmingham	29	1	2.27	28	3.27
Dundee	29	0	0.00	29	3.39
Nottingham	26	0	0.00	26	3.04

Table 1 also compares the medical schools of origin of psychiatrist and non-psychiatrist merit award-winners for the ten medical schools with the greatest numbers of award-winners; the table contrasts the distributions of psychiatrist award-winners and non-psychiatrist award-winners that the graduates of the medical schools achieved. Pearson's Chi-Square test showed a statistically significant difference between the distributions of the medical schools of origin for psychiatrist merit award-winners versus the non-psychiatric merit award-winners, *p* < 0.01.

Table 2 considers the top 10 medical schools by raw total number of award-winners, and demonstrates the effect of the approximate size correction for the medical schools on the ranking of the medical schools. London university medical schools' number one ranking for psychiatrist award-winners before size correction dropped to a number five ranking after approximate size correction. Glasgow medical school's number three ranking for psychiatrist award-winners before size correction became a number two ranking after size correction.

**Table 2:** Top 10 medical school rankings by number of graduates winning merit awards; with or without approximate size correction as of 2019–2020

Medical school	Total number of psychiatrist award-winners	Ranking by number of psychiatrist award-winners	Ranking by psychiatrist award-winners after size correction	Total number of non-psychiatrist award-winners	Ranking by number of non-psychiatrist award-winners	Ranking by non-psychiatrist award-winners after size correction
London	9	1	5	170	1	7
Edinburgh	6	2	1	78	3	2
Glasgow	5	3	2	108	2	1
Aberdeen	4	4	3	56	4	4
Cambridge	2	5	4	41	6	6
Birmingham	1	6	6	28	9	10
Oxford	0	-	-	45	5	3
Nottingham	0	-	-	26	10	9
Dundee	0	-	-	29	8	5
Manchester	0	-	-	38	7	8

The methodology we applied in this analysis compared tier 3, tier 2 and tier 1 award-winners in psychiatry. Our data demonstrate that the tier 1 psychiatrist award-winners stemmed from two medical schools: Sheffield and Glasgow. In comparison, the psychiatrist award-winners in tier 2 came from a wider background of six medical schools: London university medical schools, Glasgow, Edinburgh, Cambridge, Harvard and Bangalore. Finally, the award-winners in tier 3 stemmed from a yet wider background of 17 medical schools. These were Zimbabwe, Orange, Newcastle, Malta, London university medical schools, Leeds, Kerala, Ireland, Glasgow, Edinburgh, Danylo Halytsky Lviv, Cambridge, Bristol, Bombay, Birmingham, Alexandria and Aberdeen.

The data depicted in Table 3 permits comparison of the geographical locations of the medical schools of origin by continent and number of award-winners; for both psychiatrists and non-psychiatrists. 84.1% of the psychiatrist award-winners originated from European medical schools. In contrast, 92.1% of the non-psychiatrist award-winners originated from European medical schools. The distributions of continental locations of medical school origins of psychiatrist and non-psychiatrist award-winners was shown to be statistically significant by a Pearson's Chi-Square test, *p* < 0.01.

**Table 3:** A geographical comparison of the medical schools of origin of psychiatrist and non-psychiatrist merit award-winners as of 2019–2020

Continental location of medical school	Non-Psychiatrists		Psychiatrists	
	Total number of non-psychiatrist award-winners	Percentage of total number of non-psychiatrist award-winners	Total number of psychiatrist award-winners	Percentage of total number of psychiatrist award-winners
Europe	787	92.1	37	84.1
Asia	38	4.44	3	6.82
Africa	16	1.87	3	6.82
North America	4	0.47	1	2.27
Australasia	9	1.05	0	0
South America	1	0.12	0	0
Total	855	100%	44	100%

We designated UK and Irish medical schools as local institutions and so were able to identify international medical graduates (IMGs). 22.7% of the psychiatrist award-winners were international medical graduates, whereas 10.8% of the non-psychiatrist award-winners were IMGs. *The IMGs showed the greatest representation in the tier 3 category of award-winners where they represented 24.2% of the psychiatrist award-winners.*

Table 4 represents a raw ranking for each recorded clinical specialty by number of award-winning doctors. Table 5 represents a ranking of *aggregated* similar specialties by number of award-winning doctors.

**Table 4:** Raw rankings for stated specialties by number of award-winners as of 2019–2020

Specialty Ranking	Number of award-winners	Specialty Ranking	Number of award-winners	Specialty Ranking	Number of award-winners
1. Medicine	185	19. Academic GP	10	37. Blood Transfusion	2
2. General Medicine	102	20. Geriatric Medicine	10	38. Clinical Radiology	2
3. Surgery	88	21. Emergency Medicine	9	39. Endocrinology & Diabetes	2
4. Anaesthetics	61	22. Orthopaedic Surgery	8	40. Forensic Psychiatry	2
5. Paediatrics	48	23. Cardiothoracic Surgery	6	41. Infectious Diseases	2
6. Pathology	39	24. Chemical Pathology	6	42. Plastic Surgery	2
7. Public Health Medicine	34	25. Clinical Genetics	6	43. Psychiatry of Learning Disability	2
8. General Surgery	30	26. Communicable Diseases	6	44. Respiratory Medicine	2
9. Obs and Gynaecology	26	27. Dermatology	6	45. Anaesthetics	1
10. Psychiatry	24	28. ENT Surgery	6	46. Cardiology	1
11. Dental	23	29. General Practice	6	47. Occupational Medicine	1
12. Ophthalmology	19	30. Paediatric Surgery	6	48. Public Health Dentistry	1
13. Diagnostic Radiology	16	31. Urology	6		
14. General Psychiatry	16	32. Clinical Oncology	5		
15. Haematology	16	33. Genito Urinary Medicine	4		
16. Histopathology	16	34. Oral and Maxillofacial Surgery	4		
17. Neurology	14	35. Medical Microbiology & Virology	2		
18. Radiology	14	36. Acute internal Medicine	2

**Table 5:** Rankings of combined specialty-groups by number of Distinction/Excellence award-winners

Specialty-Group Rankings	Number of award-winners	Percentage of award-winners
1. Medical disciplines	337	37.5%
2. Surgical disciplines	224	24.9%
3. Laboratory medicine disciplines	95	10.6%
4. Anaesthetics	62	6.90%
5. Paediatric disciplines	48	5.33%
6. Psychiatric disciplines	44	4.89%
7. Public health medicine	34	3.78%
8. Radiological disciplines	32	3.56%
9. GP disciplines	16	1.78%
10. Dermatology	6	0.667%

## Discussion

### Psychiatrist merit awards and UK medical schools

Our study is part of the first series to comprehensively analyze British clinical merit award-winners' medical schools of origin. This project identifies medical schools that have facilitated the successful medical education of psychiatrists by using the outcome measure of clinical merit award-winning. As a result, the data and analysis we provide will be of significance to local potential medical students as well as current and future graduates of International Medical Programs [14]. Our series of studies are the first to *rank medical schools by the number of merit award-winners* originating from each school, and accordingly will provide a new perspective for medical educators.

The UK has been known to attract international medical graduates to practice medicine. This was further confirmed and quantified in the General Medical Council 2019 workforce study that stated "For the first time, more non-UK medical graduates took up a licence to practise than UK medical graduates [15]." As a result of such workforce migrations, the scope of possible medical schools of origin of merit award-winners has essentially become global. Specifically, our database of merit award-winners covering the 2019–20 round has 85 different medical schools represented. This study shows that after being chosen by a "transparent and defensible" assessing and scoring arrangement [16] 59.1% of the psychiatrist award-winners received their undergraduate training at one of only five UK medical schools (Table 1). In ascending order, these were Cambridge, Aberdeen, Edinburgh, Glasgow and London. A similar pattern of concentration occurred amongst the non-psychiatrist merit award-winners; 53.4% of these were graduates of Oxford, Aberdeen, Edinburgh, Glasgow or London university medical schools. The observation that there is a similar concentration of award-winners amongst graduates of similar medical schools, for both the psychiatrists and non-psychiatrists, implies that there may be common underlying non-specialty specific factors which account for the success of these doctors. The quality of undergraduate medical education could be such a factor.

Our data showed that Aberdeen and Edinburgh medical schools were statistically more likely to be represented in the psychiatric group of award-winners than the non-psychiatrist award-winners. One possible explanation may relate to higher suicide rates in Scotland drawing the attention of medical schools to the potential importance of psychiatric training and psychiatric services, in aiding their local communities.

Because the top medical schools of origin for psychiatrist award-winners include London university medical schools and Cambridge, then for these schools good quality of medical education and prestige would seem to coincide [17]. Interestingly and in contrast, Aberdeen is also highly ranked amongst the psychiatrist award-winners and suggests that a notably prestigious medical school alone is not the dominant factor in the career success of these award-winning clinicians. The rankings of medical schools that we produced in this study provide data which future prospective medical students can use to select medical schools appropriate for their ambitions. Students generally make rational decisions in the field of education [18, 19] and ranking information of this type is particularly important to an educational pathway as complex and tortuous as training to be a doctor in any given specialty. Recent studies have demonstrated that the differences between medical schools tend to remain stable [20], so the guidance offered here will have valuable longevity.

Our observation of the concentration of award-winning psychiatrists and non-psychiatrists amongst a small number of medical schools probably indicates a role for size consideration. Specifically, after summation of the number of yearly graduates, London medical schools combine to be one of the largest medical schools in Europe. Therefore, as a proportion, London university medical schools' graduates would probably be well represented in any essentially Eurocentric merit award schemes. To investigate this, we performed an approximate size correction to the medical school rankings by number of award-winners, as described and discussed in the methods section, using the 2019 medical school student admission numbers. Applying this to the psychiatrist award-winners rankings, London university medical schools dropped from a position of one before the approximate size correction to a position of five after size correction. A parallel effect occurred when the approximate size correction was applied to the non-psychiatrist award-winning rankings; here London university medical schools dropped in ranking from one to seven. Clearly, medical school size affects the medical school ranking. However, it is improbable that size alone can account for the concentration of clinical merit award-winners in a few medical schools; a factor related to the quality of the undergraduate medical education is consistent with our findings.

### Psychiatrist merit awards and international medical schools

The medical schools of origin of award-winners were also analyzed by continental location, this being pertinent to the travel and relocation of medical professionals in the modern era of globalization [21, 22]. The continental medical schools of origin of the psychiatrist and non-psychiatrist award-winners were compared (Table 3). The vast majority of psychiatrist and non-psychiatrist merit award-winners were trained in European medical schools (87.1% and 93.2%, respectively). A comparison of the continental distributions of the medical school origins of award-winners showed that there was a statistically significant difference between psychiatrist and non-psychiatrist award-winners. Specifically, psychiatrist award-winners were more likely to have trained in medical schools in Africa or Asia than their non-psychiatrist award-winning colleagues. As psychiatry is perceived to be a low status discipline that has usually been undersubscribed in UK training deaneries and is acknowledged by the Royal College of Psychiatrists as having too few NHS consultants, this profession may be seen as a less competitive entry point into British medicine for medical workforce migrators seeking socioeconomic improvement. In contrast, the more local European medical graduates may see the low status of psychiatry as a more important factor in their career choice than the economic considerations.

Our study shows a greater diversity of medical school origin amongst the lowest grade of national merit award-winners than the highest grade of national merit award-winners. Psychiatrists with tier 1 awards came from two medical schools representing just one continent whereas tier 2 award-winners came from six medical schools representing three continents. Tier 3 award-winners originated from 17 medical schools representing four continents. These findings appear to represent a tendency to greater globalization and inclusivity effects in the lower national merit awards. The fact that the greatest concentration of IMGs occurred amongst the lowest national merit awards also supports this observation. The greater number of lower grade awards and the shorter time taken to attain the lower awards than the higher awards, would naturally make such demographic trends more apparent amongst the lower merit awards. Longitudinal analyses of merit award-winners over the next decade would be valuable in accurately assessing whether this diversity trend progresses into the higher merit awards.

### Postgraduate psychiatrist training and merit awards

The established and longstanding recruitment crisis into psychiatry training has resulted in the filling rates for postgraduate **c**ore **t**raining entry (**CT**1) in the UK being historically low [23, 24]. Accordingly, the low numbers of psychiatrist award-winners displayed in Tables 4 and 5 are in part due to the low numbers of UK trained psychiatrists. Unfortunately, this would also have the negative feedback effect of decreasing the number of award-winning psychiatrists to act as role models/mentors for trainees that might themselves have become award-winners. Taking steps to lessen the recruitment crisis should eventually increase the number of psychiatry award-winners and break this vicious cycle. This process may already have begun with the 100% filling rates in the British core training posts in 2022 [25]. Of course, a more rapid way to break this cycle would involve the award schemes themselves directly increasing the number of psychiatrist award-winners, to bring them closer to parity with similar clinical specialties (Tables 4 and 5).

Poor medical school training in psychiatry has itself been implicated in the recruitment crisis [24]. Our identification of the medical schools most likely to contribute to the production of successful psychiatrists, *also identifies positive sources of trainees that are more likely to do well in core training* as well as providing educational models for the less successful medical schools. If it is desirable for trainee doctors to learn from good clinicians, then it would also seem as desirable for the less successful medical schools to learn from the more successful medical schools. Better medical school training in psychiatry will assist in the amelioration of the recruitment crisis by inspiring future core training candidates.

Our data show that the large number of IMGs in psychiatry trainee posts (44%) [26] feeds through into the number of IMGs in the psychiatrist merit award-winners (22.7%) although these are mainly concentrated in the lowest national merit awards. Future studies may demonstrate that IMGs redistribute more evenly amongst the tier 1 and tier 2 merit award-winners. These award-winners are inevitably perceived as leaders in their fields and so whether IMGs or non-IMGs, they tend to extend the frontiers of academic and clinical psychiatry that have benefits throughout the specialty. Such overt and visible examples of leadership [27] are important for trainees to see in individuals that they can identify with and follow.

### Merit awards; undergraduate and postgraduate training of psychiatrists and non-psychiatrists

Our study is unique in directly relating national merit award winners in psychiatry (and non-psychiatry) to their medical schools of origin. In fact, there is very little comprehensive research that relates UK medical schools to their individual performances in training medical students and the subsequent postgraduate performances of their students. Of the three studies we identified [20, 28, 29] the most comparable to ours was the MedDifs project by McManus et al*.* [20] In that substantial project they studied the differences in UK medical school performances by aggregating data from 50 measures, both quantitative and qualitative, that were categorized as applicant selection, institutional history, curricular influences, student satisfaction, teaching/learning and assessment, foundation year one perception, foundation phase entry scores, postgraduate examination performance, specialty training choice and fitness to practice. Clearly and in contrast, our study was limited in not analyzing as many educationally related factors as well as not employing a qualitative research approach. Accordingly, the MedDifs study was able to compare the relationships and note both positive and negative correlations between their large number of measures (*e.g.* Problem Based Learning school graduates producing lower scores in postgraduate exams, graduates of larger medical schools tending to perform worse in their postgraduate exams and alumni of schools with greater self-regulated learning performing better in postgraduate exams). However, the MedDifs study was less able to describe the likely causal relationships between its measures. Both of our projects had the similar limitation of being unable to comparatively evaluate the medical schools at the level of specific courses within their undergraduate programs.

In order to investigate the possible causalities of our presented medical school rankings for psychiatrist and non-psychiatrist merit award-winners, we reviewed the histories of the UK medical schools [30–39]. We noted that all seven of the oldest medical schools, measured by establishment date, in the UK were present in the top 10 medical school rankings of the combined total merit award-winners. These were all established prior to 1826 and consisted of Birmingham (1825), Manchester (1824), Aberdeen (1786), St Bartholomew's university (1785), Glasgow (1751), St George's London University (1733) and Edinburgh (1726) medical schools. Moreover, Oxford medical school was known to have been teaching medicine since the twelfth century and Cambridge had been teaching medicine since 1524; in essence, these two medical schools had been teaching clinical disciplines before the formal establishment process had even been formed. Accordingly, it can be stated that of the top 10 medical school rankings for combined total merit award-winners, *8 are the oldest medical schools in the UK.*

Furthermore, none of the more modern medical schools (established after 1999) are represented in our top 10 medical school rankings by total number of merit award-winners. So, Warwick (2000), Norwich (2000), Peninsula (2000), Brighton and Sussex (2002), Hull York (2003), Keele (2003) and Swansea (2004) are not represented our top 10 (or top 20) medical school award-winner rankings. Whilst it may be understandable that the younger medical schools established within the last ten years may not yet have had time for their alumni to distinguish themselves to merit award levels, it is less clear that this explanation accounts for the dearth of top 10 ranked medical schools established around the year 2000.

**Fig. 1:**
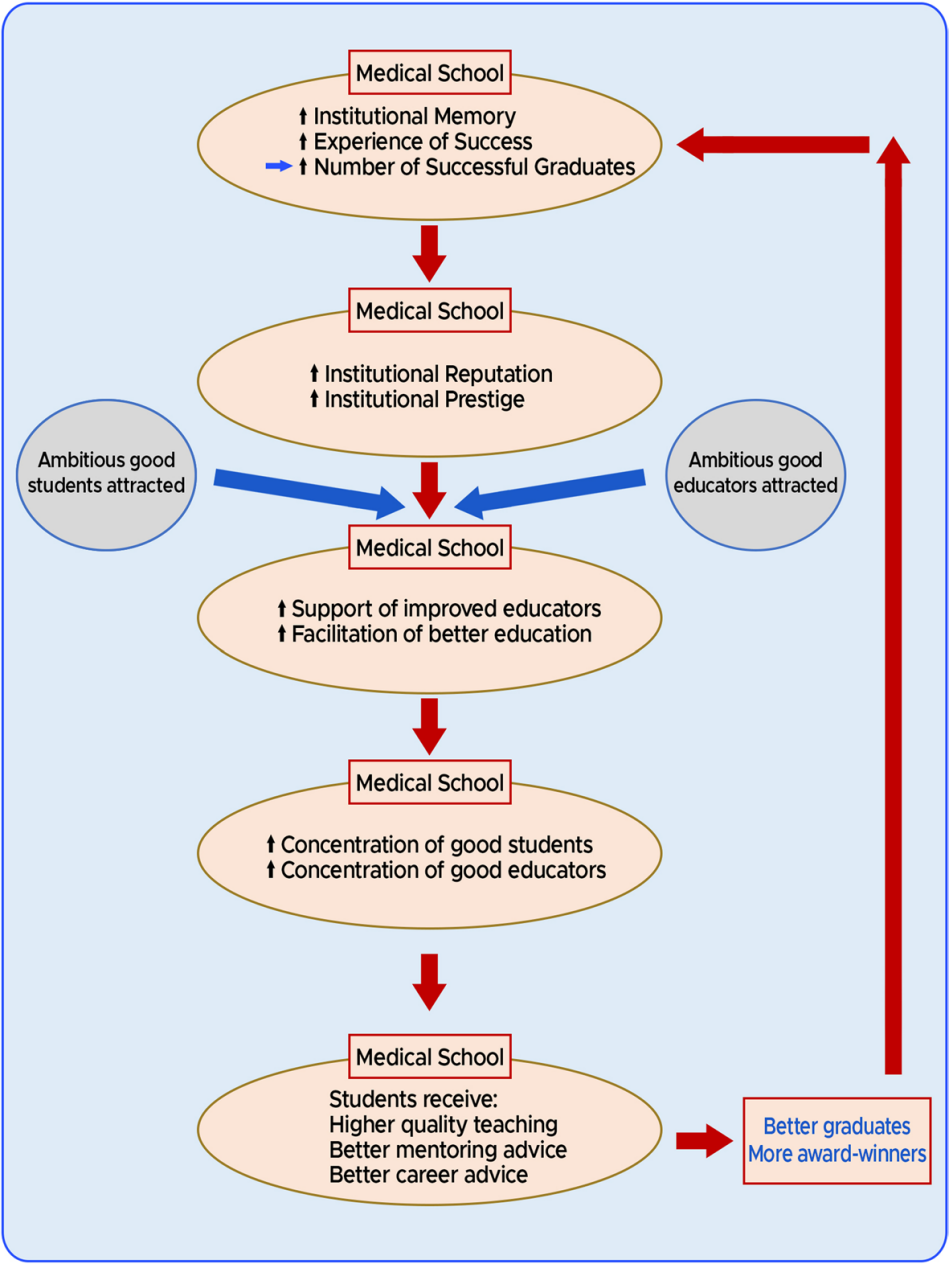
A model for the creation of award-winners. Cycles of institutional memory and experience

*In summary, our observations are consistent with at least a correlation between medical school age and the number of subsequent graduates becoming merit award-winners.* After considering the totality of the results of our research study and also accepting the previous results of the studies into UK medical school education [20, 28, 29], in Fig. 1 we reiterate a model we first proposed earlier this year [13]—a model accounting for the age dependent differential medical school performance in creating award-winning doctors:

### Cycles of institutional memory and experience

1. As a result of their greater longevity, the older medical schools have more institutional memory and experience in education than the younger medical schools. So, the older medical schools have a greater chance of producing successful alumni before the younger schools have even been established.
2. Because of the older medical schools apparently greater number of visibly successful alumni, they may appear more prestigious with better institutional reputations. Accordingly, ambitious and able students are more likely to be attracted to these medical schools.
3. These older medical schools with greater institutional memories and experience of producing students who achieved better postgraduate outcomes, are better placed to use this background knowledge to support and facilitate better educators and better education.
4. Therefore, these medical schools will accumulate a greater proportion of more able students and more able educators.
5. Then, the students in these university medical schools are more likely to benefit from higher quality teaching, better mentoring and better career advice.
6. Consequently, these medical schools are more likely to generate better prepared alumni who have a greater chance of becoming merit award-winners. *The training of these successful doctors will add to the institutional memory and increase the medical school's successful experience in education and so the cycle will repeat.*

It should be understood that the older medical schools will necessarily have had more time to undergo more repeats of this cycle, creating a cumulative effect and thus increasing the number of successful merit award-winners originating from their schools. We also suggest that part of the reason for the differences between medical school educational performances may stem from the relative effectiveness of this cycle in different medical schools. *Moreover*, *it should also be noted that the same studies which apply to the generation of this cycle of institutional memory and experience, also apply at the faculty/departmental levels. In the case of psychiatrists, a faculty or department that produces award-winning psychiatrists is more likely to produce more award-winning psychiatrists in the future. In essence, this would be a positive feedback cycle of faculty/departmental memory and experience.*

Any award scheme designed and administered by human beings runs the risk of introducing biases, thus leading to overrepresentation by particular groups. Our model provides a natural explanation and mechanism for connecting excellence/success with such bias. With every cycle of our model, increasing numbers of successful graduates originating from the older universities accumulate in the UK medical community. Subsequently, such distinguished and visible alumni are more likely to be elevated to senior leadership or managerial positions. These positions would include clinical excellence/distinction award allocators. Consequently, explicit selection biases or implicit selection biases would have a tendency to favour the graduates of these same medical schools of origin—resulting in a disproportionate number of these alumni gaining awards. Ultimately, we believe our model of *Cycles of Institutional Memory and Experience,* at least in part accounts for the concurrence of appropriate success/excellence in award-winning and apparent bias in our medical school rankings. Accordingly, it seems inevitable that the effects of genuine appropriate award attainment and bias are linked and would be expressed simultaneously.

In the last year there has been a reorganization of the UK national clinical excellence scheme. Specifically, in January 2022, it was announced that the latest iteration would be termed the "National Clinical Impact Awards, NCIA [40]." The governing authority announced that the objectives of this scheme would be to:

- Widen access.
- Simplify the application process, attempting to make it more equitable and inclusive.
- Reward excellence in a wider range of activities and behaviours [41].

This new rewards scheme offers a natural test and challenge to our *Cycles of Institutional Memory and Experience* model. Our model is based on the history and epidemiology of medical education in the UK. Accordingly, an analysis of the medical schools of origin of the NCIA winners should yield similar rankings to those reported in our series of publications, assuming that there is an underlying value to the model. We look forward to testing our model in this way.

## Conclusions

By using merit awards as outcome measures, our study contributes original medical education data to the pool of information that describes the demographic distribution of successful psychiatry clinicians in Britain. Specifically, we identify the medical schools that are most associated with the production of award-winning psychiatrists. We also identify the medical schools that are most associated with the production of award-winning non-psychiatrists. *We are the first to produce a ranking of medical schools by the number of psychiatrist merit award-winners.* We provide evidence for a rational choice of medical education centres for ambitious psychiatrically inclined, non-psychiatrically inclined and undecided students.

We demonstrate that international medical graduates are making substantial contributions to good psychiatric clinical practice in Britain, as judged by their concentration amongst the lower national merit award-winners. We provide evidence that indicates globalization and diversity of medical school origin are being reflected in the merit awards, indicating that Britain is a credible destination for ambitious medical trainees that seek national or international success.

## Data Availability

Data from this article is available upon reasonable request to the authors. Dr Steele is the corresponding author and will make the data available. https://www.sehd.scot.nhs.uk/publications/DC20200319SACDA.pdf; https://www.gov.uk/government/publications/accea-annual-report-2020; https://www.gmc-uk.org/registration-and-licensing/the-medical-register; https://olr.gdc-uk.org/SearchRegister

## Abbreviations

IMG: International Medical Graduate
CT: Core Training
NCIA: National Clinical Impact Awards
CEA: Clinical Excellence Awards
DA: Distinction Awards
